# The heparan sulfate 3-*O*-sulfotransferases (HS3ST) 2, 3B and 4 enhance proliferation and survival in breast cancer MDA-MB-231 cells

**DOI:** 10.1371/journal.pone.0194676

**Published:** 2018-03-16

**Authors:** Charles Hellec, Maxime Delos, Mathieu Carpentier, Agnès Denys, Fabrice Allain

**Affiliations:** University of Lille, CNRS, UMR 8576—UGSF—Unité de Glycobiologie Structurale et Fonctionnelle, Lille, France; University of South Alabama Mitchell Cancer Institute, UNITED STATES

## Abstract

Heparan sulfate 3-*O*-sulfotransferases (HS3STs) catalyze the final maturation step of heparan sulfates. Although seven HS3ST isozymes have been described in human, 3-*O*-sulfation is a relatively rare modification, and only a few biological processes have been described to be influenced by 3-*O*-sulfated motifs. A conflicting literature has recently reported that HS3ST2, 3A, 3B and 4 may exhibit either tumor-promoting or anti-oncogenic properties, depending on the model used and cancer cell phenotype. Hence, we decided to compare the consequences of the overexpression of each of these HS3STs in the same cellular model. We demonstrated that, unlike HS3ST3A, the other three isozymes enhanced the proliferation of breast cancer MDA-MB-231 and BT-20 cells. Moreover, the colony forming capacity of MDA-MB-231 cells was markedly increased by the expression of HS3ST2, 3B and 4. No notable difference was observed between the three isozymes, meaning that the modifications catalyzed by each HS3ST had the same functional impact on cell behavior. We then demonstrated that overexpression of HS3ST2, 3B and 4 was accompanied by increased activation of c-Src, Akt and NF-κB and up-regulation of the anti-apoptotic proteins survivin and XIAP. In line with these findings, we showed that HS3ST-transfected cells are more resistant to cell death induction by pro-apoptotic stimuli or NK cells. Altogether, our findings demonstrate that HS3ST2, 3B and 4 share the same pro-tumoral activity and support the idea that these HS3STs could compensate each other for loss of their expression depending on the molecular signature of cancer cells and/or changes in the tumor environment.

## Introduction

Heparan sulfate (HS) is a highly sulfated glycosaminoglycan found at the cell surface and in the extracellular matrix. It mediates cell-cell and cell-matrix communications and regulates the binding of a large number of ligands, resulting in a variety of physiological and pathological effects, such as in embryonic development, cell growth and differentiation, homeostasis, inflammatory response, tumor growth and microbial infection. These interactions are driven at a first electrostatic level by the overall sulfation of HS, and then by the specific recognition of structural determinants, especially the specific arrangement of sulfate groups in a given sequence [1,2].

The structural diversity in HS is derived from enzymatic modifications of the nascent polymer composed of alternating D-glucuronic acid (GlcUA) and *N*-acetylated D-glucosamine (GlcNAc) units. This non-sulfated precursor is first subject to partial *N*-deacetylation/*N*-sulfation of GlcNAc residues, which leads to the occurrence of consecutively *N*-sulfated regions (S-domains), regions that escape modification and remains *N*-acetylated, and regions of transition alternating *N*-acetylated and *N*-sulfated units. The further modifications include C5 epimerization of some GlcUA into L-iduronic acid (IdoUA), 2-*O*-sulfation of uronic acid residues -mainly IdoUA- and 6-*O* and/or 3-*O*-sulfation of GlcN units. These modifications are catalyzed by HS maturation enzymes, including *N*-deacetylases/*N*-sulfotransferases (NDST), C5-epimerase and 2-*O*, 6-*O* and 3-*O*-sulfotransferases (HS2ST, HS6ST, HS3ST). To date, four NDSTs, three HS6STs and seven HS3STs have been cloned and characterized in human. These isozymes are expressed in a different way depending on cell type and tissue environment, which renders the schema of HS maturation more complex [2,3]. Although HS3STs represent the largest family of HS sulfotransferases, the reaction of 3-*O*-sulfation is the rarest modification found within HS compared to the widely distributed *N*-, 6-*O*-GlcN and 2-*O*-UA sulfations. Moreover, it is the last modification in HS biosynthesis, which requires that the substrates for HS3STs have been already modified at other positions by NDSTs, HS2ST and C5-epimerase [4,5]. HS3ST1 was described to transfer a sulfate group to the 3-OH position of *N*-sulfated GlcN (GlcNS) residue that is linked to a non-sulfated GlcUA residue at the non-reducing side, while HS3ST5 exhibits a broader substrate specificity and transfers a sulfate group to GlcNS linked to either GlcUA or IdoUA irrespective of 2-*O*-sulfation. Both these isozymes are critically involved in the generation of anticoagulant-active HS/heparin domains, as they participate in the synthesis of the binding site for antithrombin-III (AT-III). In contrast, HS3ST2, 3A, 3B, 4 and 6 introduce a sulfate group to the 3-OH position of GlcNS residue that is linked to an adjacent upstream 2-*O*-sulfated IdoUA. These isozymes, which are also called “gD-type” HS3STs, were reported to provide the HS-binding site for the protein gD of type I herpes simplex virus (HSV-1) and to assist viral entry [6–12]. It is also worth noting that hundreds HS-binding proteins have been identified, but only a little number of ligands are known to have a specific affinity for 3-*O*-sulfated HS sequences [5,13]. In this context, compared to the other HS modifications, 3-*O*-sulfation emerge as a structural determinant insuring selective interactions.

The functions of 3-*O*-sulfated HS in the regulation of physiological and pathological processes have gained a growing interest in recent years. In addition to its well-defined role in the anti-coagulant properties of HS/heparin, 3-*O*-sulfation has been reported to play a critical role in cancer. These assumptions are based on the observations that tumor cell behavior is dramatically altered by the expression of gD-type HS3STs. As examples, HS3ST2 was reported to be critically involved in breast cancer cell invasiveness [14]; high expression of HS3ST3B induces epithelial-mesenchymal transition in pancreatic cancer cells [15] and promotes the proliferation of acute myeloid leukemia cells [16]; the pathological expression of HS3ST4 plays a deleterious role in the escape of cancer cells to the immune system [17]. On the other hand, a conflicting literature has suggested that some of these isozymes may exhibit anti-oncogenic properties. Aberrant methylation of the genes encoding HS3ST2 and HS3ST3A was indeed described in various cancers. Reversing methylation restored the expression of both these HS3STs and resulted in the suppression of tumor cell growth [18–21]. More recently, HS3ST3A was described as a tumor regulator in the development of breast cancer, with contrasting anti-oncogenic or tumor-promoting effects depending on the phenotype of cancer cells [22]. Although contradictory, all these studies suggest that abnormal expression of certain gD-type HS3STs may have a functional impact on cancer cell behavior. However, the effects of individual expression of one isozyme compared with the others and the molecular mechanisms underlying these effects remain still largely unknown. Hence, we decided to evaluate the consequences of an overexpression of HS3ST2, 3A, 3B and 4 in the same cellular model. To this end, we carried out a comparative study of the responses induced by the expression of each isozyme in the breast cancer cell line MDA-MB-231. Overall, we found that HS3ST2, 3B and 4 enhance cell proliferation and promote efficient protection against cell death, which suggests that the three isozymes may display a prominent role in breast cancer progression.

## Materials and methods

### Materials

Rabbit antibody to HS3ST2 and mouse antibody to HS3ST3A (clone E-12) were obtained from Thermo Fisher Scientific (Waltham, MA, USA) and Santa-Cruz Biotechnology (Santa Cruz, CA, USA), respectively. Mouse antibodies to HS3ST3B, HS3ST4 and survivin were purchased from R&D Systems (Minneapolis, MN, USA). Antibodies to phospho-Akt^(S473)^, Akt, phospho-c-Src^(Y416)^, c-Src, phospho-ERK1/2^(T202/Y204)^, phospho-STAT3^(Y705)^, STAT3, I-κB, phospho-NF-κB p65^(S536)^, NF-κB p65, XIAP and secondary antibodies conjugated to horseradish peroxidase (HRP) were purchased from Cell Signaling Technology (Danves, MA, USA). Mouse antibody to GAPDH was from Santa Cruz. HS4C3 and MBP49 antibodies were provided by T. Van Kuppevelt (University of Nijmegen, Netherlands) and used as described in [23]. Other chemicals and antibodies, including anti-ERK1/2, were from Sigma-Aldrich (Darmstadt, Germany) unless otherwise specified.

### Construction of expression plasmids

The cDNA sequences encoding human HS3ST2 (NM_006043) and HS3ST3A (NM_006042) were obtained from primary macrophages, while the encoding region for HS3ST3B (NM_006041) was isolated from the fibroblast MRC-5 SV2 cell line [24,25]. Each cDNA was amplified by PCR with specific forward and reverse primers (Table 1) and DynaZyme DNA Polymerase (Thermo Fisher Scientific), as described by the manufacturer. PCR fragments were generated with *NheI* or *XhoI* restriction sites at the 5’ end and *XhoI* or *BamHI* at the 3’ end. After digestion with the appropriate restriction enzymes, fragments were inserted in pcDNA3.1. Expression plasmid encoding HS3ST4 (NM_006040) was constructed as described in [17] and provided by J. Cherfils-Vicini (University of Nice, France). Subsequently, the coding DNA sequence (CDS) was inserted in pcDNA3.1 using *NheI* and *XhoI* restriction sites. Each construct was sequenced by GATC Biotech AG (Constance, Germany) to verify the cDNA sequence and the insert positions.

**Table 1:** Sets of primers used for plasmid construction. The underlined sequences represent restriction sites for the generation of PCR fragments.

Enzymes	Forward (F) and reverse (R) primers
HS3ST2	F: 5’-atatGATATCgccaccatggcctatagggtcctgggccgcg-3’ R: 5’-gtccAAGCTTttattcccacctgaagtcctgcccaac-3’
HS3ST3A	F: 5’-ATATCTCGAGGCCACCATGGCCCCTCCGGGCCCGGCCAGRG-3’ R: 5’- GTCCGGATCCTTATCCATCCCAGCCAAAGTCGTGCCCGGTC -3’
HS3ST3B	F: 5’-TTAACTCGAGATGGGGCAGCGCCTGAGTGGCGGCAGATCTTGCCTCGAT-3’ R: 5’- GAGCGGATCCTCAATCCCAGCCAAAGTCGTGCCCGGTCATCTGGTAGAA -3’

### Cell culture and transfection

The human breast cancer cell lines MDA-MB-231 cells (ATCC® HTB-26™) and BT-20 cells (ATCC® HTB-19™) were routinely cultured in Dulbecco’s Modified Eagle Medium (DMEM) supplemented with 10% fetal calf serum (FCS) (Lonza, Verviers, Belgium), at 37°C in an atmosphere containing 5% CO_2_. Before use, cells were plated at 2×10^5^ cells per well and transiently transfected with complexes containing expression plasmids and lipofectamine 2000 (1 μg in 4 μL), according to the manufacturer’s instructions (Thermo Fisher Scientific). After transfection, cells were cultured in DMEM supplemented with 1% FCS.

### RNA isolation and real-time RT-PCR

Total RNA was isolated from 2×10^5^ cells using the NucleoSpin RNA II kit, according to the instructions of the manufacturer (Macherey-Nagel, Düren, Germany). Reverse transcription was performed from 1 μg of total RNA by using the Maxima First Strand cDNA Synthesis Kit for RT-qPCR (Thermo Fisher Scientific). Synthetic primers for HS3ST3A, HS3ST3B and HS3ST4 were described in [24]. Synthetic primers for HS3ST2 were designed by using Primer-Blast (NCBI): 5’-GCTCTCGAGGGTCCTGGGCA-3’ (forward), 5’-TGTGGGCGTGAAGAAGGGGG-3’ (reverse). Specificity of the primers was checked by semi-quantitative RT-PCR on a 2.5% (w/v) agarose gel. All of them amplified only one fragment of expected size, for which the sequence was confirmed (GATC Biotech, Constance, Germany). Real-time PCR amplifications were performed using an Mx3000P Multiplex Quantitative PCR system (Agilent Technologies, Santa Clara, CA, USA), as described in [26]. The transcript of HPRT was used as a control to normalize the expression of our genes of interest. The amplification efficiency of each primer pair was performed on serial dilutions of cDNA. The point at which the PCR product was first detected above a fixed threshold, termed cycle threshold (*Ct*), was determined for each sample, and the average *Ct* of triplicate samples was used for analysis.

### SDS-PAGE and Western blot

MDA-MB-231 cells (4×10^5^ per point) were lysed in 150 μL of lysis buffer (50 mM Tris-HCl, 150 mM NaCl, 1% Triton X-100, 0.1% SDS, pH 8.0) supplemented with a mixture of protease and phosphatase inhibitors (Roche Diagnostics, Meylan, France) for 3 h at 4°C. Lysates were clarified by centrifugation at 10,000 g for 30 min at 4°C. Protein content of the supernatants was estimated using micro-BCA protein assay kit (Thermo Fisher Scientific). Samples corresponding to twenty micrograms of proteins were mixed with Laemmli buffer and boiled for 10 min. Proteins were then separated by SDS-PAGE and transferred onto nitrocellulose membrane (Amersham, Uppsala, Sweden). The membrane was blocked for 1 h at room temperature in 20 mM Tris-HCl, pH 7.6, 150 mM NaCl (TBS) with 0.05% (v/v) Tween-20 and 5% (w/v) BSA (Roche), and then probed with primary antibodies (1/2000) overnight in TBS supplemented with 5% (w/v) BSA. After washing, HRP-conjugated secondary antibodies (1/10,000) were added for 1 h at room temperature and immunoreactive proteins were detected using ECL prime Western blotting detection (GE Healthcare). Quantification of immunostaining intensity was performed by using Image J software.

### Compositional analysis of HS disaccharides

Composition of HS was analysed by reverse phase-high performance liquid chromatography (RP-HPLC), using a fluorescent method of pre-column labelling of disaccharides with 2-aminoacridone (AMAC), as described in [24,27]. Briefly, 30 x 10^6^ cells were collected and treated with Pronase E (Merck Millipore, Darmstadt, Germany) (1.5 mg/ml) and benzonase (250 mU/ml). After clarification, samples were loaded on DEAE-Sepharose column (Merck Millipore). The column was extensively washed with phosphate buffer containing 0.3 M NaCl, after which remaining bound molecules were eluted with the same buffer containing 2 M NaCl. Chloroform was then added to the sample (vol/vol) and the mixture was stirred vigorously. Aqueous phase was recovered and dialysed against water for 16 h at 4°C (Slide-A-Lyser 2000 Da, Thermo Fischer Scientific). After freeze drying, material (5 μg of total glycosaminoglycans, as quantified by carbazole assay) was treated with a mixture of heparinases I, II and III (Iduron, Manchester, UK) (10 mU each/sample) for 16 h at 37°C. Sample was then filtered on an Amicon 3000-Da unit (Merck Millipore) and the fraction containing disaccharides was collected and freeze-dried. For AMAC labelling, HS digests were dissolved in 10 μL of glacial acetic acid/DMSO (15:85, v/v) solution containing 0.1 M AMAC plus 10 μL of sodium cyanoborohydride solution (1 M in water). The reaction was carried out for 5 h at 45°C, after which time 15 μL of 50% (v/v) DMSO was added to the samples. AMAC-labelled disaccharides were then diluted with water to a final volume of 200 μL and applied to a Kinetex core-shell C18-column (Phenomenex, Le Pecq, France) equilibrated in 60 mM ammonium acetate, pH 5.6 (eluent A), running on a Varian Prostar 260 HPLC system. After a 7 mL gradient of 0–10% solution B (100% methanol), disaccharides were eluted over a 35 mL linear gradient of 10–30% solution B at a flow rate of 0.7 mL/min. Disaccharides were detected by in-line fluorescence (excitation at 428 nm and emission at 525 nm), using a Varian ProStar 363 detector, and data were analysed with the Star software (v6.43).

### Cell proliferation

MDA-MB-231 or BT-20 cells were plated at 5×10^4^ cells/mL in 4 mL of DMEM, transfected with expression plasmids and then cultured for 24 h or 48 h. Then, they were collected and directly counted with Trypan Blue to exclude dead cells. In parallel experiments, cell proliferation was estimated by using the Cell-Titer 96 Aqueous Non-Radioactive Cell Proliferation Assay kit (Promega, Fitchburg, USA). To this end, 2×10^3^ transfected cells were plated in 200 μL. At the indicated times, 20 μL of MTS/PMS (95:5, v/v) solution was added to each well of culture and reaction was developed at 37°C for 1 h. Absorbance was measured at 490 nm using a BioTek Epoch microplate reader (BioTek Instruments, Winooski, USA). This assay involves conversion of a MTS tetrazolium compound to a colored formazan product whose absorbance is directly proportional to the number of metabolically active cells, thus providing information on the number of viable cells in cell proliferation experiments.

### Colony formation assay

To investigate the effects of each HS3ST on colony formation, 2×10^3^ transfected MDA-MB-231 cells were seeded in six-well plates and cultured in DMEM complemented with 1% FCS for 9 days and then in DMEM with 10% FCS for 3 days. At the end of culture, cells were washed with phosphate buffer saline (PBS), fixed in the presence of 4% paraformaldehyde in 0.1 M sodium phosphate buffer (pH 7.2) for 30 min at room temperature, and stained with 0.05% crystal violet for 15 min. After extensive wash with water, the number of colonies was counted.

### Induction and measurement of apoptosis

Cell apoptosis was evaluated by measuring phosphatidylserine externalization and caspase-3 activation, essentially as described in [26]. Briefly, apoptosis was induced in transfected MDA-MB-231 cells (2×10^5^ cells per point) by the addition of 100 ng/mL of anti-Fas antibody (Merck Millipore) plus 100 ng/mL TNF-α (PeproTech) for 24 h at 37°C, or by the addition of 1 μM staurosporin (Merck Millipore) for 4 h at 37°C. Thereafter, cells were stained with annexin-V and propidium iodide (PI) by using the Annexin-V Apoptosis Detection Kit I (BD Biosciences) and analysed by flow cytometry using a FACSCalibur instrument and the CellQuest software (BD Biosciences). This procedure allows assignment of cells in viable (annexin-V^−^/PI^−^), early apoptotic (annexin-V^+^/PI^−^), late apoptotic (annexin-V^+^/PI^+^) and necrotic (annexin-V^−^/PI^+^) populations. Apoptosis was also evaluated by measuring the activation of caspase-3, by using the Caspase-3 assay fluorimetric kit (Sigma-Aldrich). For each experimental condition, 5×10^4^ cells were lysed overnight at -80°C and the enzymatic activity of caspase-3 was measured thereafter by incubation of cell lysates with Ac-DEVD-AMC substrate. Specificity of the reaction was checked by the addition of Ac-DEVD-CHO caspase-3 inhibitor. After 60 min of incubation at 37°C, release of AMC product was monitored at 405 nm by using a fluorimeter microplate reader (CentroLuminometer, Berthold, Bad Wildbad, Germany). Values of absorbance were converted in caspase-3 activity by using an AMC standard curve and expressed in nanomoles of AMC released per min and per mL of cell lysate.

### Isolation and culture of NK cells

Human citrated venous blood samples were obtained from the local blood transfusion center (Lille, France). Following isolation of peripheral blood mononuclear cells by density centrifugation on Lymphoprep (Eurobio-AbCys, Courtaboeuf, France), NK cells were purified with magnetic beads coupled to CD56, according to the instructions of the manufacturer (BD Biosciences, San Jose, CA, USA). Purity of the cell population was assessed by flow cytometry and found > 95%. NK cells were then cultured for 24 h in RPMI 1640 medium supplemented with 10% (v/v) heat-inactivated FCS, in the presence of 50 μg/mL of poly-IC (polyinosinic-polycytidylic acid sodium salt) (Sigma). The experiments were undertaken with the understanding and written consent of each donor (EFS, NT/18/2015/092). The study methodologies conformed to the standards set by the Declaration of Helsinki and were approved by the local ethics committee (French research ministry, DC-2008-242).

### Measurement of NK cell cytotoxicity

NK cell-induced cytolysis of MDA-MB-231 cells was analysed by measuring the activity of released lactate dehydrogenase (LDH) in cell supernatants, using the CytoTox 96^®^ Non-Radioactive Cytotoxicity Assay kit (Promega, Fitchburg, USA). Briefly, 10×10^3^ transfected MDA-MB-231 cells were plated in 100 μL of PBS complemented with 2.5% FCS. NK cells were then added into the wells at the target cells/effector cells ratios 1:1, 1:5, 1:10 and incubated for 5 h at 37°C in the presence of 20 ng/mL of interferon-γ (IFN-γ). Thereafter, 50 μL of the supernatants are mixed with 50 μL of LDH substrate and incubated for 30 min in the dark. Absorbance was measured at 490 nm as above. Maximum LDH release was induced by the addition of 0.8% Triton X100. The percentages of cytotoxicity were determined as follows: [(experimental lysis–effector spontaneous lysis–target spontaneous lysis) / (target maximum lysis–target spontaneous lysis)] × 100.

### Statistical analysis

Results are representative of at least three independent experiments conducted with separate preparations of transfected cancer cells and NK cells from different donors. All values are expressed as the means ± SD. Statistical significance between the different values was analysed by using one-way ANOVA and two-tailed Student’s *t*-tests, with a threshold of *P* < 0.05 considered as significant.

## Results

### Overexpression of HS3ST2, 3A, 3B and 4 in breast cancer cells

**Fig 1:**
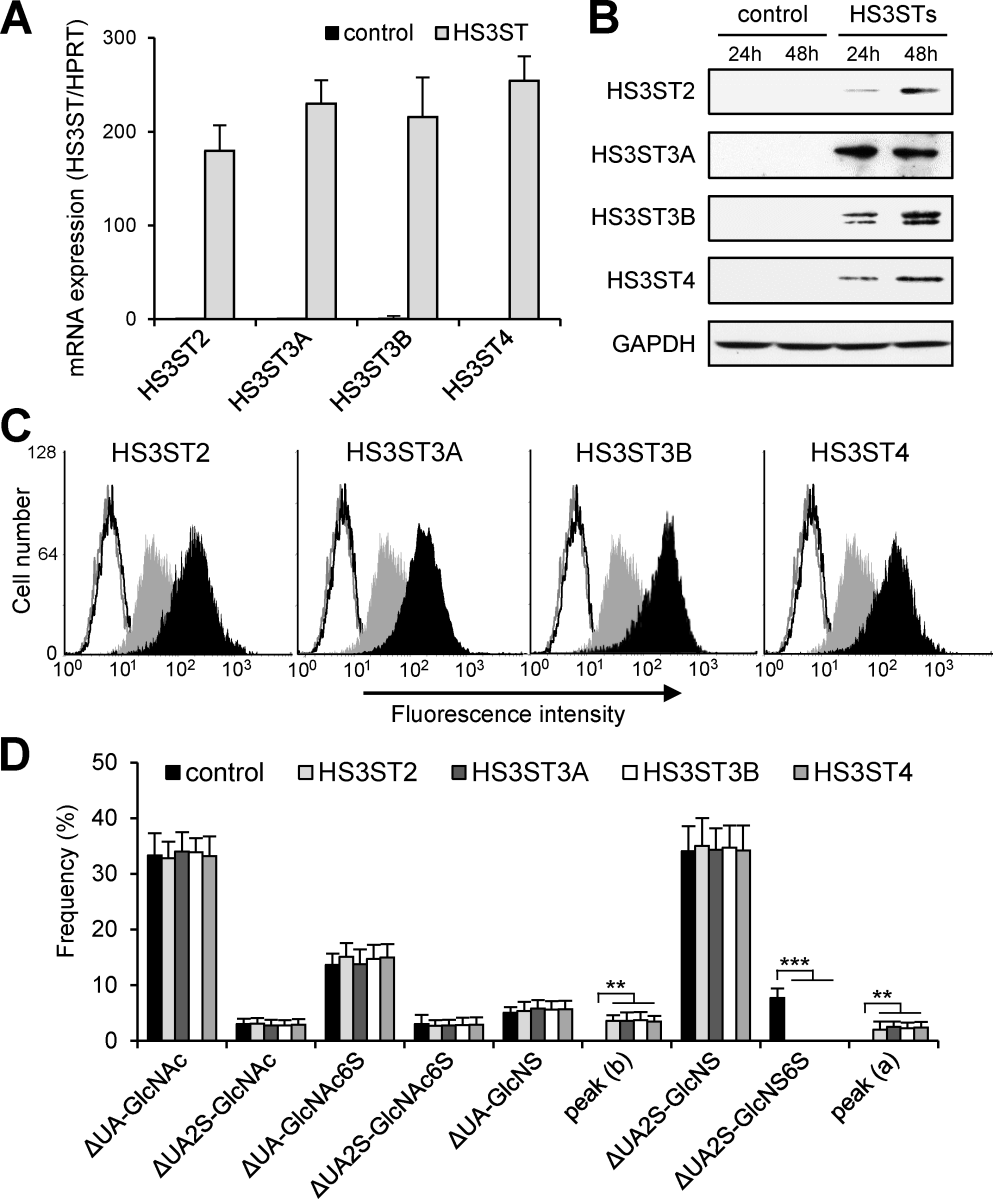
Overexpression of gD-type HS3STs in MDA-MB-231 cells. Cells were transiently transfected with empty vector (control) or vectors encoding HS3ST2, HS3ST3A, HS3ST3B or HS3ST4, and then cultured for 24 h and 48 h prior analysis of the expression and activity of each isozyme. (**A**) Following RNA extraction, the mRNA levels of HS3STs were quantified by real-time RT-PCR at 24 h post-transfection. Relative abundance of the transcripts was normalized to endogenous HPRT mRNA. Data are means ± SD from at least three distinct experiments. (**B**) In parallel experiments, proteins from cell lysates were separated by SDS-PAGE and subjected to Western blotting with appropriate specific antibodies. Parallel immunoblotting with antibodies to GAPDH confirmed equal loading of the samples. (**C**) Efficiency in the reaction of heparan 3-*O*-sulfation was confirmed 48 h post-transfection by using HS4C3 antibody. Cell surface fluorescent staining was detected by flow cytometry. Filled histograms represent the staining obtained with HS4C3 antibody, while open histograms correspond to the binding of the irrelevant antibody MBP49. Each panel represents the binding of the antibodies to the surface of HS3ST-expressing cells (black histograms) compared to the binding of the same antibodies to control cells (grey histograms). Data are representative of three separate experiments. (**D**) Compositional disaccharide analysis of HS from transfected MDA-MB-231 cells. Following depolymerization of HS with a mixture of heparinases, disaccharides were labelled with AMAC and resolved by RP-HPLC (see S1 Fig). Data are expressed as frequency of each disaccharide and correspond to means ± S.D. from two sets of triplicate injections performed with distinct cell preparations (**P < 0.01, ***P < 0.001, significantly different when compared to control cells).

The expression of type gD HS3STs is either very low or negligible in epithelial tumor cells, when compared to the other sulfotransferases involved in HS maturation [14,22,23]. In order to examine the functional impact of high levels of expression of these enzymes in cancer cell behavior, we decided to use transient overexpression systems. MDA-MB-231 was chosen as a representative breast cancer cell line of the basal-like subtype [28]. As expected, transfection with the plasmids encoding HS3ST2, 3A, 3B and 4 resulted in a strong expression of each isozyme in MDA-MB-231 cells, as demonstrated by RT-PCR (Fig 1A) and Western blot (Fig 1B). Flow cytometry analysis with anti-HS epitope antibody indicated that overexpression of each gD-type HS3ST was accompanied by an increase in 3-*O*-sulfation of cell surface HS. When compared with parental cells, we found indeed that the expression of each HS3ST enhanced the binding of HS4C3 antibody (Fig 1C), which preferentially reacts with highly sulfated HS epitopes containing 3-*O*-sulfate groups [23].

In order to provide structural information on the changes induced by HS3ST overexpression in MDA-MB-231 cells, HS were purified and digested with a mixture of heparinases. The resulting disaccharides were labelled with AMAC and analysed by RP-HPLC with fluorescent detection (S1 Fig). As already described [24,27], the fluorescence profiles contained identifiable peaks, which are representative of the typical HS components. Analysis of HS digests from MDA-MB-231 cells transfected with empty vector revealed an overall level of sulfation of ∼1.2 sulfate/disaccharide, coupled to relative high amounts of ΔUA-GlcNAc (33 ± 4%) and ΔUA2S-GlcNS disaccharides (34 ± 4%). In contrast, it did not contain a great abundance of the trisulfated ΔUA2S-GlcNS6S disaccharide (7 ± 2%) (Fig 1D). Analysis of HS digests from HS3ST-overexpressing cells also indicated a composition dominated by ΔUA-GlcNAc and ΔUA2S-GlcNS disaccharides. The overall sulfation level was estimated to ∼1.17 sulfate/disaccharide on average, without any notable difference between each HS3ST-overexpressing cell type. However, a striking observation was the complete disappearance of the trisulfated ΔUA2S-GlcNS6S disaccharide in the elution profiles of HS digests. Conversely, analysis of HS components revealed the appearance of new peaks, which may be 3-*O*-sulfated products (S1 Fig). Due to the unavailability of commercial standards, 3-*O*-sulfated disaccharides cannot be directly identified. In their work, Mochizuki *et al*. [29] reported that gD-type HS3STs mainly use IdoUA2S-GlcNS6S-containing sequences as acceptor substrates and produce tetrasulfated units as a major product from both heparin and HS. Consequently, the disappearance of the trisulfated disaccharides could reflect the reaction of 3-*O*-sulfation in HS3ST-overexpressing cells. Most notably, the peak named (a) in the elution profile of HS digests from HS3ST-overexpressing cells could be the tetrasulfated disaccharide containing a 3-*O*-sulfo group. On another hand, previous works reported the presence of unusual 3-*O*-sulfated GlcNH_3_^+^ residue in heparin [30,31]. Nevertheless, such 3-*O*-sulfated *N*-unsubstituted GlcN units have never been characterized in natural HS, which suggest that they could derive from *N*-desulfation during the process of purification and/or depolymerisation [5,29]. Thus, the small peak (b) that elutes just after the ΔUA2S-GlcNS standard may contain such products containing 3-*O*-sulfated GlcNH_3_^+^ units (Fig 1D).

### Effects of HS3ST overexpression on cellular proliferation and viability

**Fig 2:**
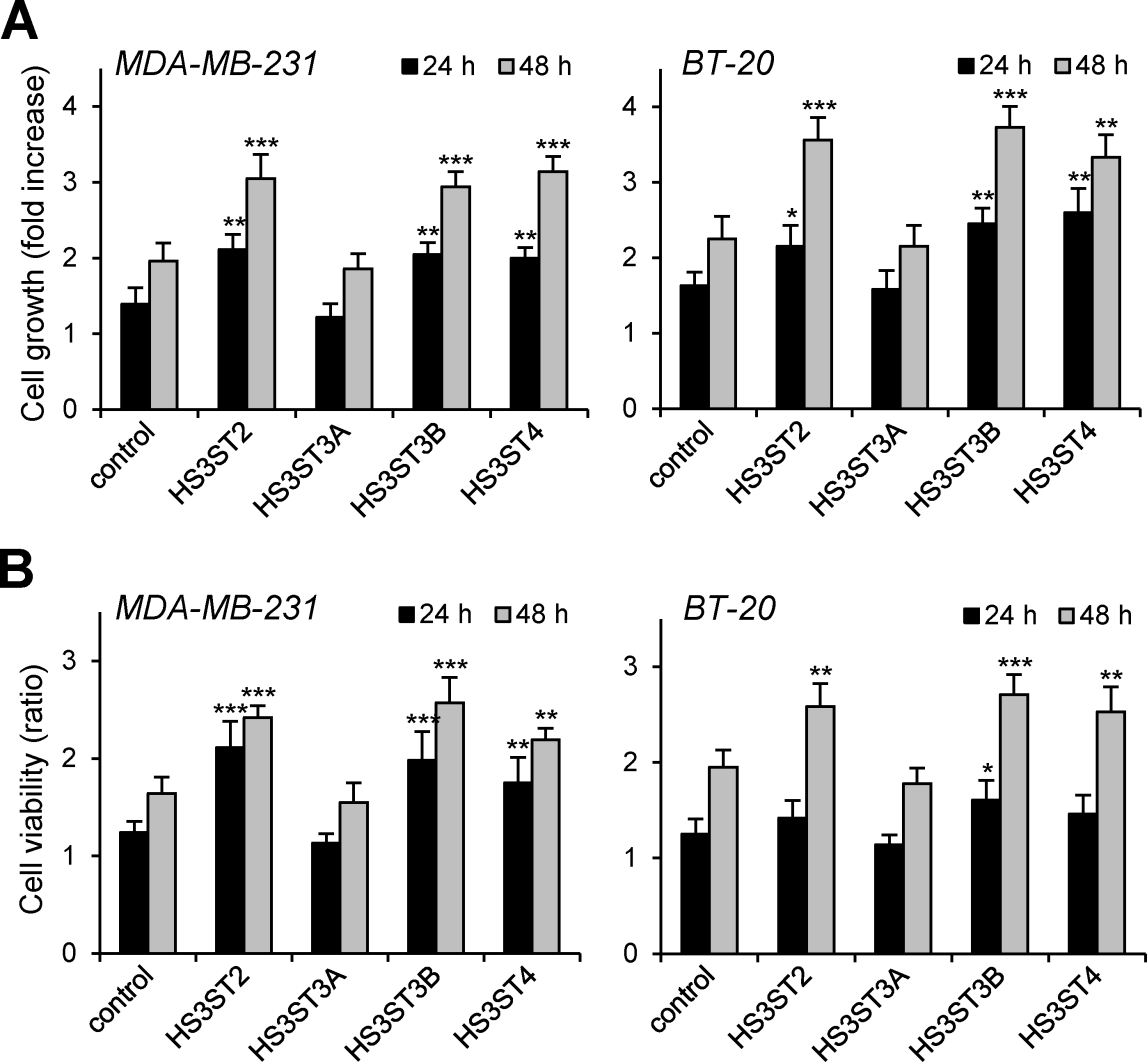
Effects of the expression of HS3ST2, HS3ST3A, HS3ST3B and HS3ST4 on breast cancer cell growth. MDA-MB-231 and BT-20 cells were transfected with overexpression vectors and cultured in medium containing 1% FCS for 24 h and 48 h. At each time, the effects of HS3STs on the growth of MDA-MB-231 and BT-20 cells was estimated by cell counting (**A**) and MTS assay (**B**). Results are expressed as fold changes by comparison with control cells transfected with empty vector. Data are means ± S.D. from three separate experiments performed independently (*P < 0.05, **P < 0.01, ***P < 0.001, significantly different when compared to control cells).

In previous works, HS3ST2 was reported to increase the viability and invasiveness of MDA-MB-231 breast cancer cells [14]. More recently, overexpression of HS3ST3A was described to have an opposite effect on the proliferation of MDA-MB-231 cells [22]. Thus, we decided to reevaluate the effects of these gD-type HS3STs in the same cellular model. To this end, MDA-MB-231 and the less invasive BT-20 cells were transiently transfected with plasmids encoding HS3ST2, 3A, 3B or 4, and thereafter cultured for 24 and 48 h in the presence of 1% of FCS. In our hands, overexpression of HS3ST2 resulted in a significant increase in the growth of both cancer cell lines. At 48 h post-transfection, the rates of proliferation (Fig 2A) and viability (Fig 2B) of HS3ST2-transfected MDA-MB-231 cells were about 1.6- and 1.4-fold increased as compared with parental cells transfected with vector control, respectively. A similar increase in the rates of proliferation and viability of BT-20 cells was also observed (×1.6 and ×1.3 as compared to vector control, respectively). Interestingly, we found that the expression of HS3ST3B and HS3ST4 similarly enhanced the proliferation and viability of both cell lines, without any notable difference between the three isozymes. In contrast, overexpression of HS3ST3A had no significant impact on the proliferation of MDA-MB-231 and BT-20 cells, meaning that HS3ST3A did not share the same properties than other gD-type HS3STs. Together, these first results suggest that the impact of HS3STs on cancer cell behavior could be dependent on the type of isozyme.

**Fig 3:**
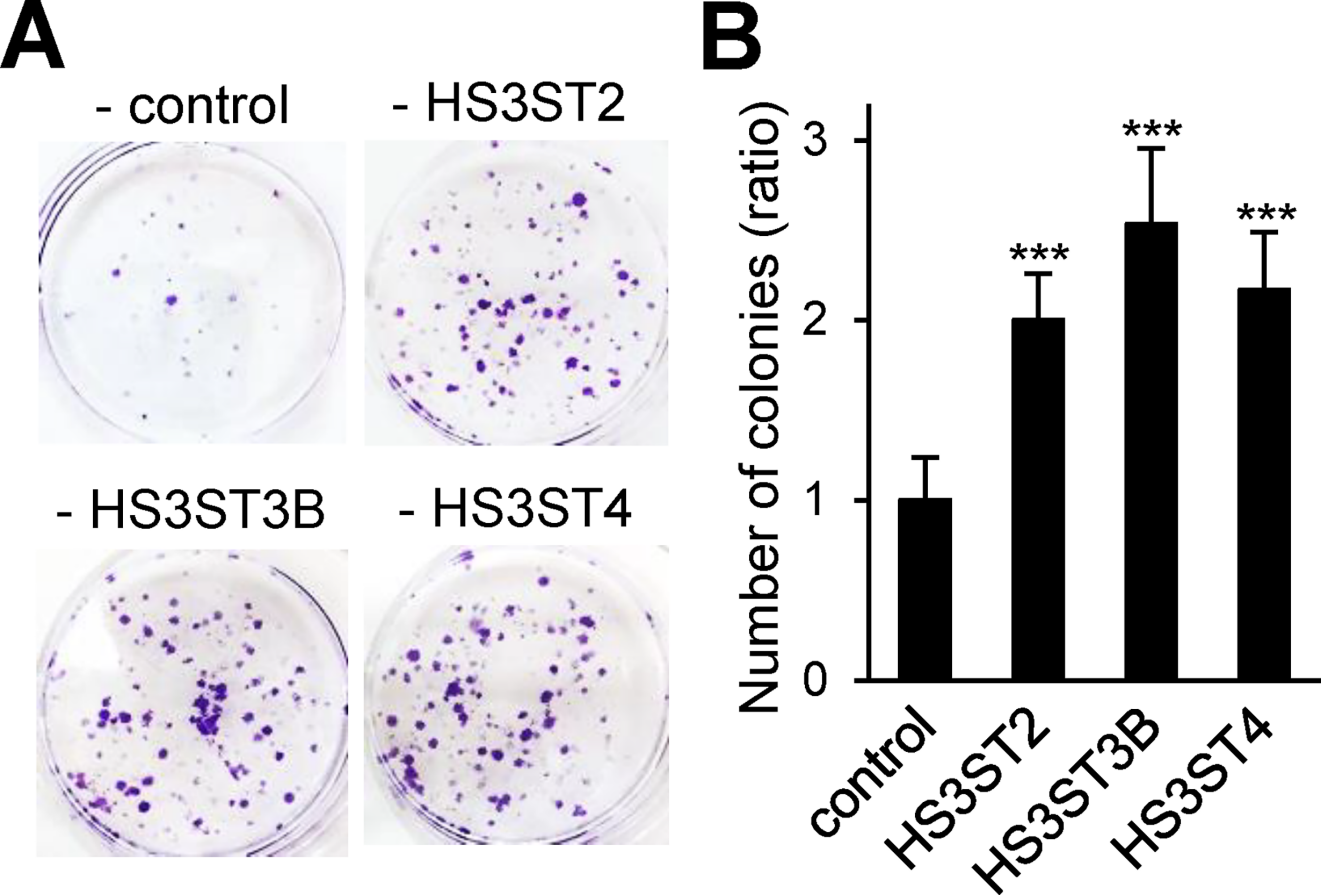
Effects of the expression of HS3ST2, HS3ST3B and HS3ST4 on colony formation in MDA-MB-231 cells. (**A**) Equal numbers of control and HS3ST-overexpressing cells (2000 per well) were seeded in six well plates and maintained for nine days in DMEM complemented with 1% FCS to form colonies. Fresh complete growth medium was then added for three days, after which the colonies were stained with crystal violet. The right panel (**B**) represents the quantification of the colonies per well. Results are expressed as fold changes by comparison with control cells transfected with empty vector. Data are means ± S.D. from five separate experiments performed independently (***P < 0.001, significantly different when compared to control cells).

When the experiments were reproduced in the presence of 10% FCS, we did not observe any notable difference in cell growth between control and HS3ST-expressing cells. These findings suggest that overexpression of HS3ST2, 3B or 4 probably enhanced cell proliferation and viability via the activation of autocrine signaling pathways, which would be masked by the presence of 10% FCS. To test this hypothesis, clonal survival assays were carried out with MDA-MB-231 cells. Such an experiment could give us important information about the ability of individual cells to grow into colonies. Transfected cells were thus seeded at low cellular density (2×10^3^ cells per well) and cultured in the presence of 1% FCS. Thereafter, colonies were stained with crystal violet and counted (Fig 3A). Our results showed that the colony forming capacity of MDA-MB-231 overexpressing HS3ST2, 3B and 4 has more than doubled when compared to control cells, and no notable difference was observed between the three isozymes (Fig 3B). In contrast, overexpression of HS3ST3A had no enhancing effect on the colony forming capacity of MDA-MB-231 cells, as compared with parental cells transfected with control vector (S2 Fig). Together, these results reinforce the idea that HS3ST2, 3B and 4 have the same functional impact on the proliferation and viability of MDA-MB-231 cells.

### Modulation of signaling pathways by the expression of HS3STs

**Fig 4:**
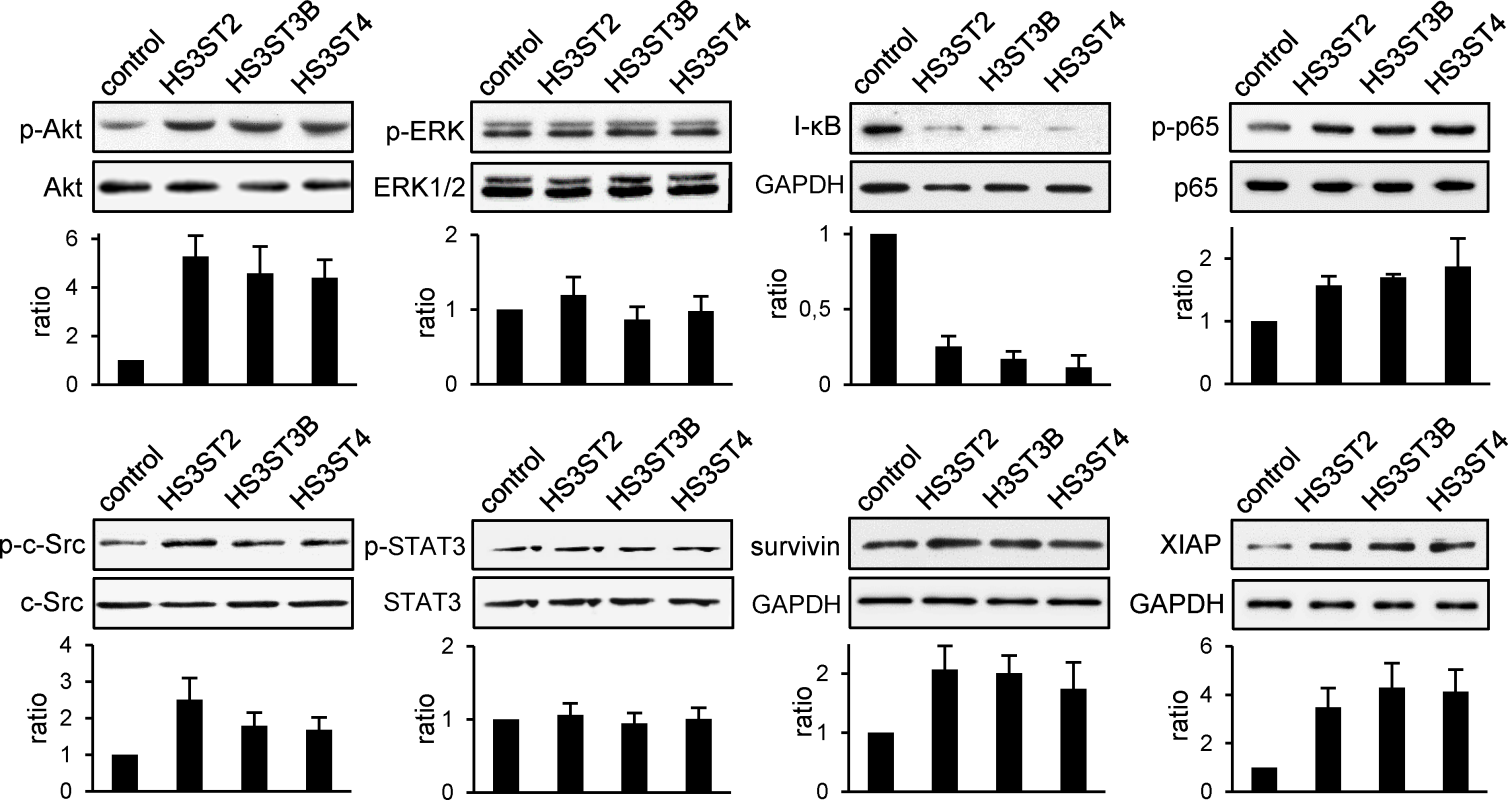
Effect of the expression of HS3ST2, HS3ST3B and HS3ST4 on cell signaling. Twenty hours post transfection, MDA-MB-231 cells were serum-starved for 3 hours, collected and lysed. Proteins were then separated by SDS-PAGE and subjected to Western blotting with antibodies to I-κB, survivin, XIAP and to the phosphorylated forms of c-Src, Akt, ERK1/2, STAT3 and NF-κB p65 subunit. Parallel immunoblotting with antibodies to GAPDH and to c-Src, Akt, ERK1/2, STAT3 and NF-κB p65 regardless of their phosphorylation status confirmed equal loading of samples. Histograms represent the quantification of the phosphorylation status of c-Src, Akt, ERK1/2, STAT3, NF-κB p65 and of the expression of I-κB, survivin and XIAP related to GAPDH. Data were normalized to control cells. Representative results from three independent experiments are shown.

To explore the molecular mechanisms responsible for the functional impact of HS3ST2, 3B and 4, we analysed the principal signaling pathways that regulates breast cancer cell growth, invasiveness and survival [32–37]. Following transfection, MDA-MB-231 cells were cultured for 20 h in the presence of 1% FCS and then serum-starved for 3 h before analysis of the activation status of Akt, ERK1/2, c-Src, NF-κB and STAT3. As shown in Fig 4, HS3ST-transfected groups with HS3ST2, 3B and 4 all displayed higher levels of phosphorylated forms of Akt, c-Src and NF-κB p65 as compared to vector control. Concomitantly, we observed a dramatic decrease in the amount of I-κB, which confirmed the activation of the NF-κB pathway. In contrast, the levels of phosphorylated ERK1/2 and STAT3 were not significantly affected by the overexpression of HS3ST2, 3B and 4. Consistent with our first results, we found that overexpression of HS3ST3A did not have any enhancing effects on the activation of signaling pathways (S2 Fig). Sustained activation of Akt and NF-κB signaling pathways has been associated with increase in tumorigenesis and enhancement of pro-survival signals. Hence, we examined the effects of HS3STs on the expression of the anti-apoptotic molecules Bcl-2, Bcl-xL, XIAP and survivin [36–38]. Transfection with each HS3ST had no apparent effect on Bcl-2 and Bcl-xL expression, as compared to vector control (data not shown). In contrast, we found a significant increase in the levels of XIAP and survivin in transfected groups with HS3ST2, 3B and 4 (Fig 4), but not with HS3ST3A (S2 Fig). These results indicate that overexpression of HS3ST2, 3B and 4 probably enhanced cell viability by modulating certain pro-survival signals.

### HS3ST-mediated protection of MDA-MB-231 cells against apoptosis

**Fig 5:**
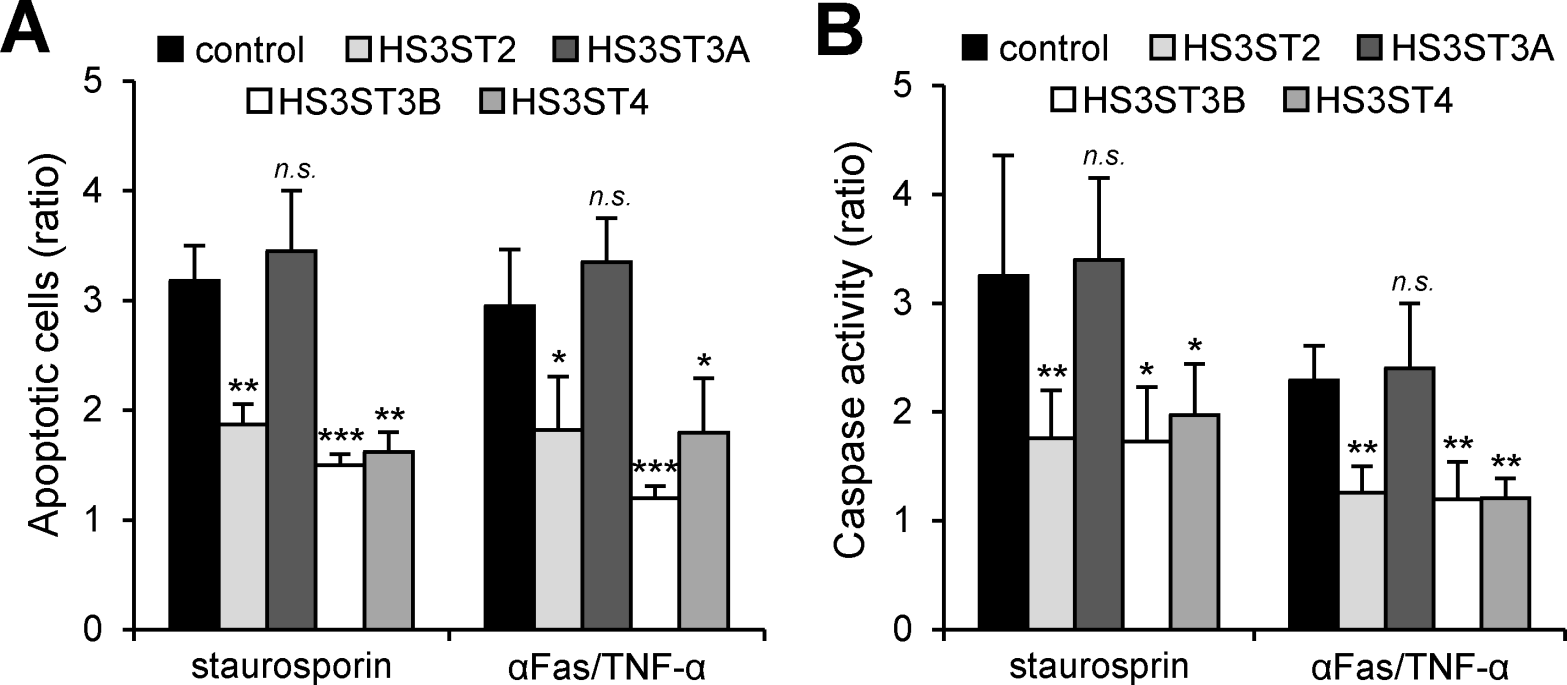
Protective effects of the expression of HS3ST2, HS3ST3B and HS3ST4 against apoptosis. Transfected MDA-MB-231 cells were treated with staurosporin (1 μM) for 5 h or with a mixture of anti-Fas antibody (100 ng/mL) and TNF-α (100 ng/mL) for 16 h. (**A**) The percentage of apoptotic cell population was evaluated by analyzing the binding of fluorescein-conjugated annexin-V. Each bar of histogram represents mean ± S.D. of the rate of apoptotic cells by comparison with untreated control cells. Data were obtained from three distinct experiments. (**B**) In parallel experiments, the activation of caspase-3 was analysed in HS3ST-overexpressing cells and in control cells after exposure to either staurosporin or to the mixture of anti-Fas antibody plus TNF-α. Results are expressed as fold increase in caspase-3 activity by comparison with untreated control cells and correspond to means ± S.D. from three independent experiments (*P < 0.05, **P < 0.01, ***P < 0.001, significantly different when compared to control cells; *n*.*s*., not significantly different).

To determine whether the increased levels of pro-survival signals in MDA-MB-231 cells may have a protective effect against apoptotic cell death, HS3ST-transfected cells were cultured for 48 h and then exposed to staurosporin for 4 h. We used this pharmaceutical agent because it strongly induces cell death via the intrinsic apoptotic pathways. In parallel experiments, HS3ST-expressing cells were also exposed to a mixture of anti-Fas antibody plus TNF-α for 24 h, in order to mimic the delivery of extracellular death signals mediated by TNF receptor and Fas antigen [39]. Analysis of cell death using annexin-V staining showed that overexpression of HS3ST2, 3B and 4 was anti-apoptotic in MDA-MB-231 cells, whereas this effect was not observed in HS3ST3A-overexpressing cells (Fig 5A). In the absence of any treatment, the percentage of apoptotic cells (including early and late apoptotic cells) was less than 10% in all groups. Exposure of control cells to staurosporin or anti-Fas/TNF-α mixture resulted in a significant induction of cell death, with an apoptotic cells rate corresponding to 32 ± 3% and 29 ± 5% of the whole cell populations, respectively. In contrast, the expression of HS3ST2, 3B or 4 significantly reduced cell death induced by both the pro-apoptotic treatments. The percentages of apoptotic cells were indeed estimated at 19 ± 2% and 18 ± 5% of the whole cell populations after exposure of HS3ST2-transfected cells to staurosporin and anti-Fas/TNF-α mixture, respectively. No notable difference was observed between the three isozymes, meaning that HS3ST3B and 4 were as effective as HS3ST2 for protecting MDA-MB-231 cells against apoptosis. In order to validate these results, we next examined the protective properties of each HS3ST on the activation of caspase-3, because of the key role of this protease in the induction of apoptosis. As shown in Fig 5B, treatment with staurosporin or anti-Fas/TNF-α mixture induced the activation of caspase-3 in control cells (×3.2 and ×2.3 compared with untreated cells). As expected, overexpression of HS3ST2, 3B and 4 was effective to reduce the activation of caspase-3, when compared to the responses measured in control and HS3ST3A-transfected cells. Collectively, these data indicate that high level of expression of HS3ST2, 3B or 4 was protective against apoptosis induced in MDA-MB-231 cells.

### HS3ST-mediated protection of MDA-MB-231 cells against NK cells

**Fig 6:**
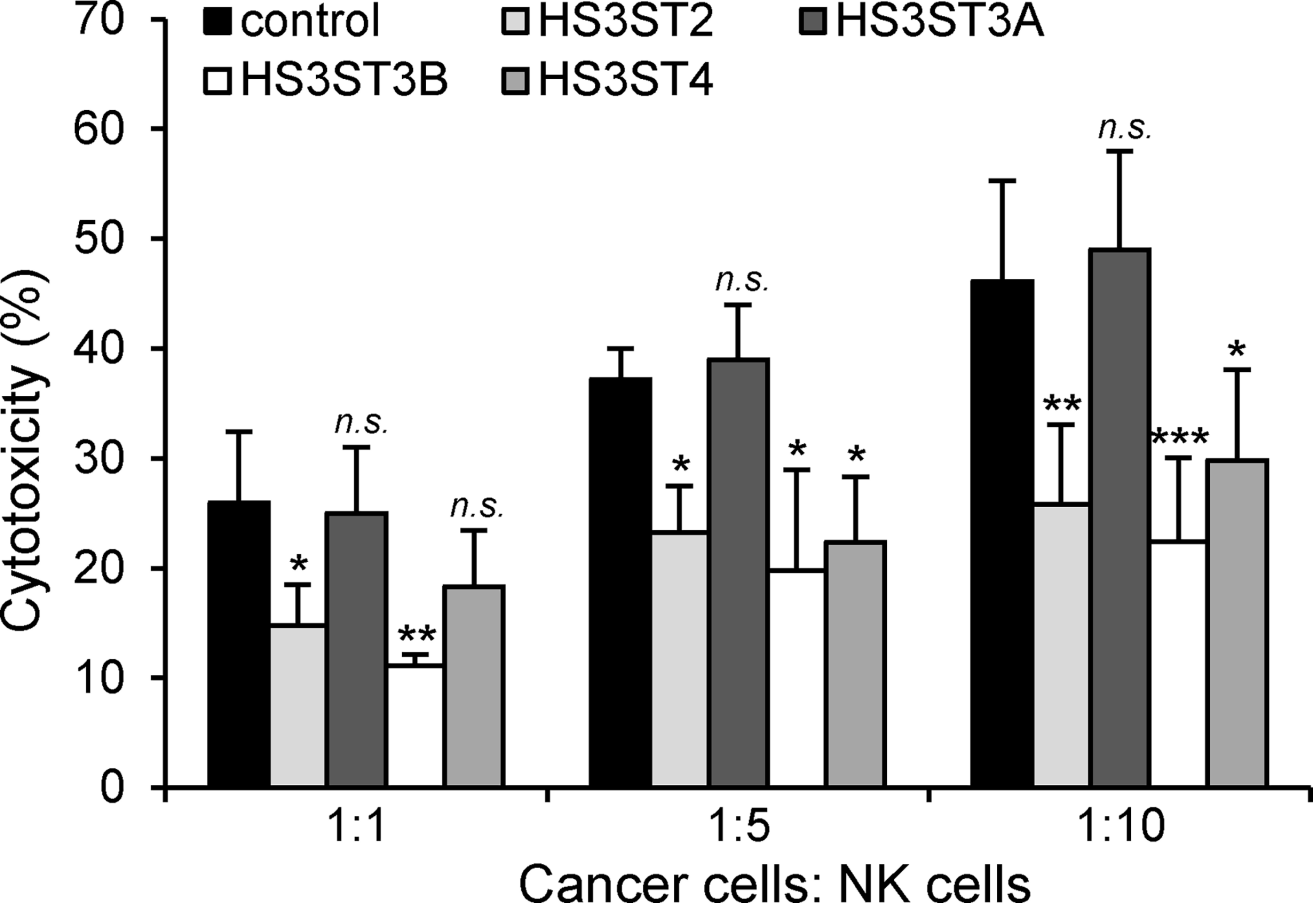
Effects of the expression of HS3STs on NK-mediated cell death. Control or HS3ST-overexpressing MDA-MB-231 cells were cultured for 32 h and then exposed to activated NK cells for 5 h at the different ratios target/effector 1:1, 1:5 and 1:10. NK-mediated cytotoxicity was estimated by using a LDH assay, as described in “Materials and Methods”. Data are means ± S.D. from three separate experiments performed with NK cells from distinct donors (*P < 0.05, **P < 0.01, ***P < 0.001, significantly different when compared to control cells; *n*.*s*., not significantly different).

The lines of evidence indicating that HS3ST2, 3B and 4 enhanced survival of MDA-MB-231 cells prompted us to investigate the impact of their expression on NK-mediated cell death. After a 5-hour incubation, the cytotoxicity induced by NK cells against HS3ST-transfected cells was detected by LDH assay (Fig 6). As expected, the lysis percentage of MDA-MB-231 cells increased along with the increase of the target/effector ratios. In control, NK cells indeed damaged 26%, 37%, and 46% of MDA-MB-231 cells at the target/effector ratios 1:1, 1:5, 1:10, respectively. Considering the same target/effector ratio, the lysis percentage of the transfected groups with HS3ST2, 3B and 4 were significantly reduced. As examples, NK cells respectively destroyed 15%, 24% and 26% of HS3ST2-transfected MDA-MB-231 cells. Apart from the differences noted with control and HS3ST3A-tranfected cells, the lysis percentages of the transfected groups with HS3ST2, 3B and 4 were not significantly different from each other, which further indicate that the three isozymes share similar protective properties in MDA-MB-231 cells.

## Discussion

The functions of 3-*O*-sulfation have been well-described in the anticoagulant activity of HS/heparin and in the facilitation of HSV-1 infection. Based on their capacity to generate binding sites for either AT-III or HSV-1 gD, HS3STs have been divided into two subgroups. HS3ST1 and HS3ST5, referred to as AT-type, are involved in the synthesis of the anticoagulant-active HS sequences, while gD-type isozymes, including HS3ST2, 3A, 3B, 4 and 6, are required to provide the HS-binding sites for the protein gD of HSV-1 [6–12]. Besides, the roles of HS3STs in the regulation of other physiological and pathological processes remains largely unclear [5]. HS3ST2, 3A, 3B and 4 have been recently described to exhibit tumor-promoting effects in various cellular models [14–17,22]. A conflicting literature has however suggested that high expression of HS3ST2 and 3A may result in the suppression of tumor cell growth [18,19,20–22]. Although contradictory, these studies suggest that abnormal expression of gD-type HS3STs may have a functional impact on cancer cell behavior. However, the function of one isozyme compared with the others and the molecular mechanisms underlying these effects remain largely unknown. Hence, we decided to compare the responses induced by transient overexpression of HS3ST2, 3A, 3B and 4 in the same cellular models.

To ascertain that overexpression of HS3STs was accompanied by changes in HS structure, we analysed the disaccharide compositions of HS from transfected MDA-MB-231 cells. As expected, we identified typical disaccharides, which are representative of the components usually found in HS. Compositional analysis of HS digests revealed however some striking differences. The most remarkable modification was the complete disappearance of the UA2S-GlcNS6S disaccharide in HS contents from HS3ST-transfected cells. These findings suggest that these trisulfated units may have served as substrate acceptors for the reaction of 3-*O*-sulfation. Two new peaks were indeed visualized in the elution profile of HS digests from HS3ST-overexpressing cells, which may correspond to 3-*O*-sulfated products [14,29]. On this assumption, 3-*O*-sulfated disaccharides would represent only a minor fraction within HS disaccharides from HS3ST-transfected MDA-MB-231 cells. These findings were however expected, because gD-type HS3STs were described to preferentially modify HS structures in S-domains, which contain trisulfated disaccharide units [5,29]. Thus, this restricted substrate specificity may explain the low frequency of 3-*O*-sulfated disaccharides in HS from gD-type HS3ST-expressing cells.

One of the principal hallmarks of cancer cells is their uncontrolled growth, which results of two complementary mechanisms: increased proliferation and establishment of survival signals giving them enhanced viability [35]. We found here that overexpression of HS3ST2, 3B or 4 resulted in a significant increase in the proliferation and viability of MDA-MB-231 and BT-20 cells, without any significant difference between the three isozymes. In their works, Vijaya-Kumar *et al*. [14] had already reported that HS3ST2 was capable of increasing the viability of MDA-MB-231 cells. In line with these findings, our results indicate that HS3ST3B and 4 share with HS3ST2 the same enhancing effects, which suggests that 3-*O*-sulfated HS may exhibit pro-tumoral properties irrespective of the HS3ST isozyme. We found, nevertheless, that overexpression of HS3ST3A did not have any enhancing effect on the proliferation and survival of MDA-MB-231 cells. Although intriguing, these results are in line with the findings that HS3ST3A rather exhibits anti-proliferative properties in MDA-MB-231 cells [22]. As a prerequisite of our study, we had checked that overexpression of each of the four HS3STs resulted in similar modifications in HS structure, which excludes that the difference observed with HS3ST3A could be due to a default in the reaction of 3-*O*-sulfation. Thus, our results suggest that the impact of gD-type HS3STs on breast cancer cell behavior could be dependent on the type of isozyme. Structural analysis showed that these isozymes recognize at least an HS pentasaccharide, in which a 2-*O*-sulfated IdoUA resides at the non-reducing side [4,11,40,41]. There is however little information about the oligosaccharide sequence towards the reducing side of the 3-*O*-sulfation sites [42]. On another hand, the diversification of HS3STs and the fact that many cell lines express multiple isozymes simultaneously suggest that there may be subtle differences in substrate specificities, even in the same subgroup. Evidence that HS3STs may generate distinct motifs has emerged from the Zebrafish model, in which two isozymes independently influence cilia function in the Kupffer's vesicle [43]. Knockdown of HS3ST5 resulted in decreased cilia length, while the loss of HS3ST6 had no impact on cilia length but impaired cilia movement. Importantly, two other HS3STs are coexpressed in the cells making up Kupffer's vesicle, but they were unable to compensate for the loss of either HS3ST5 or 6. Together, these findings demonstrate distinct roles for multiple HS3STs, supporting the idea that individual isozyme may create unique 3-*O*-sulfated sequences. In line with this assumption, HS3ST3A may have a restricted substrate specificity, making it incapable of synthesizing 3-*O*-sulfated sequences with pro-tumoral activity. Conversely, HS3ST2, 3B and 4 may exhibit a broader selectivity or share, at least in part, some common acceptors. Further investigations will be needed to better define the substrate specificities of gD-type HS3STs and to elucidate the selectivity of each isozyme.

The ability of individual cancer cells to grow into colonies is characteristic of the activation of survival signals leading to enhanced cellular viability. We found here that the colony forming capacity of MDA-MB-231 cells markedly increased after transfection with the constructs encoding HS3ST2, 3B and 4. No significant difference was observed between the three groups, which further supports the idea that high expression of each of the isozymes has the same functional impact on MDA-MB-231 cell behavior. Accordingly, we analysed the principal signaling pathways that regulates breast cancer cell proliferation and survival. In our hands, ERK1/2 and STAT3 appeared not significantly affected by the overexpression of HS3ST2, 3B and 4. A strong constitutive activation of these signaling pathways have been already described in MDA-MB-231 cells, which may explain why we did not observe any change in the levels of the phosphorylated forms of ERK1/2 and STAT3 [32,44]. In contrast, we found that overexpression of HS3ST2, 3B or 4 resulted in sustained activation of Akt, c-Src and NF-κB, in comparison with parental and HS3ST3A-transfected cells, meaning that the expression of each of the three isozymes has maintained a high activation of the corresponding signaling pathways in serum-starved cells. Aberrant activation of Akt, c-Src and NF-κB have been reported to favor tumorigenesis and to enhance the resistance to apoptosis in many cancer cell types via the activation of pro-survival signals [33–36]. In line with these observations, high levels of survivin and XIAP, two members of the Inhibitors of Apoptosis Proteins (IAP) family, were detected in MDA-MB-231 cells that expressed HS3ST2, 3B and 4. Both these anti-apoptotic proteins are highly expressed in most cancers and contribute to the protection of cancer cells by acting downstream of death receptor-induced or mitochondrial apoptosis [37,38]. These findings led us to analyse the protective effects of each of the three HS3STs against cell death induction by staurosporin or anti-Fas/TNF-α mixture. As expected, our results demonstrated that high expression of HS3ST2, 3B and 4 significantly desensitized MDA-MB-231 cells to both apoptosis-inducing treatments. Indeed, the levels of activated caspase-3 and of externalized phosphatidylserine, an indicator of mitochondrial dysfunction, were significantly reduced in transfected cells. The NF-κB pathway has been implicated in the synthesis of inhibitors of caspases, including survivin and XIAP. Both these anti-apoptotic proteins were described in turn to modulate NF-κB activation, reinforcing the idea that survivin and XIAP confer a broad advantage for cancer cells [36–38]. Accordingly, the current findings suggest that activation of the NF-κB/survivin/XIAP axis may be one of the principal mechanisms by which HS3ST2, 3A or 4 enhance cell survival. Additionally, we showed that the cytolytic activity of NK cells against cells transfected with HS3ST2, 3B or 4 was significantly reduced as compared to the lysis observed in parental or HS3ST3A-expressing cells. Exposure of cells to anti-Fas antibody plus TNF-α mimic the delivery of extracellular death signals mediated by TNF receptor and Fas antigen [39]. Thus, the protective effects of the three HS3STs against the anti-tumoral activity of NK cells could be linked to a similar inhibition of death receptor-mediated induction of apoptotic signals into target cells. On another hand, 3-*O*-sulfated HS may be also involved in the modulation of the activity of NK-cell receptors. Membrane-associated HS of target cells were indeed reported to interact with certain NK receptors [45]. Most recently, interaction between the NK receptor KIR2DL4 and 3-*O*-sulfated HS was demonstrated to regulate the activity of the receptor and its capacity to interact with other ligands [46]. Whether 3-*O*-sulfated HS of cancer cells are functional ligands for NK cell receptors remains however to be further explored.

Many HS-binding ligands use cell surface proteoglycans as co-receptors to interact with their signaling receptors, which leads to enhanced signal transduction. We showed here that transient transfection with constructs encoding each HS3ST isozyme resulted in an increase in 3-*O*-sulfated HS epitopes. Consequently, HS3ST-mediated HS alteration may have influenced ligand binding to the cell surface, leading to an alteration of diverse signaling processes. Whether 3-*O*-sulfated HS modulates ligand-receptor interactions on cancer cells is however largely unknown. On another hand, the effects of HS3ST2, 3B and 4 on cell proliferation and survival could be only observed at low concentration of FCS. These findings indicate that the functional impact of HS3ST overexpression was probably related to an autocrine mechanism of cell activation, which deserves additional consideration to identify the relevant ligands and receptors that interact with 3-*O*-sulfated motifs. To date, only a few proteins have been reported to interact with HS3ST-modified HS, probably because of the lack of available material for study [5]. Among them, cyclophilin B (CypB) was described to interact with 3-*O*-sulfated HS at the surface of T lymphocytes and monocytes/macrophages [23,31]. We recently demonstrated that it is capable of protecting macrophages against apoptosis, via a mechanism dependent on the expression of HS3ST2 [26]. HS3ST2-modified HS were also reported to enhance the binding of neuropilin-1 and to regulate its activity in neurons and endothelial cells [13]. Importantly, the expression of neuropilin-1 or its homologue neuropilin-2 by cancer cells was related to tumor initiation, growth, metastasis and immunity. They could be contributing to cell proliferation, migration, invasion and survival, by interacting with a large panel of growth factors and their cognate signaling receptors [47]. These findings support the attractive idea that HS3ST expression in cancer cells might favor the interaction of either CyPB or neuropilins with signaling receptors at the cell membrane. These assumptions are currently under investigation in our laboratory.

In conclusion, we demonstrated that overexpression of HS3ST2, 3B and 4 enhanced proliferation and survival of the breast cancer MDA-MB-231 cells. This is accompanied by increased activation of the signaling molecules c-Src, Akt and NF-κB and up-regulation of the cytoprotective proteins survivin and XIAP. Consistently, we found that HS3ST-expressing cells are more resistant to cell death induction by either pro-apoptotic stimuli or NK cells. These findings support the idea that HS3ST2, 3B and 4 share the same pro-tumoral activity in breast cancer cells. In normal physiological conditions, HS3ST3B transcripts are widely expressed in many organs. HS3ST2 was detected in neurons and macrophages, whereas the expression of HS3ST4 is restricted to the brain [4,7,24,41]. Hypermethylation of the genes encoding HS3ST2 has been described in certain cancer cells, resulting in a loss of its expression [18,20,21]. Conversely, an up-regulation of the expression of HS3ST3B has been observed in many cell types exposed to inflammatory stimuli, including monocytes/ macrophages, fibroblasts and endothelial cells [24,25,48,49]. Interestingly, we demonstrated that high expression of HS3ST3B was capable of compensating for the loss of HS3ST2 in pro-inflammatory macrophages, which means that 3-*O*-sulfated motifs could be synthesized in these cells, regardless the inflammatory status [24]. On another hand, HS3ST4 was found to be up-regulated in certain cancer cells by increased levels of TRF2, a key factor in telomere protection, which correlated with inhibition of the recruitment of NK cells to the tumor. Based on these findings, it is tempting to speculate that silenced expression of HS3ST2 could be compensated with the expression of another isozyme, *i*.*e*. HS3ST3B or 4, depending on the molecular signature of cancer cells and/or changes in the tumor environment [17]. On this assumption, our results reinforce the idea that certain gD-type HS3STs are key regulators of cancer pathogenicity and highlight the clinical value of these isozymes as future targets for therapeutic approaches.

## Supporting information

S1 Fig Disaccharide analysis of HS from HS3ST-transfected MDA-MB-231 cells. Purified HS from control (empty vector) and HS3ST-transfected cells were digested to disaccharides using a mixture of heparinases. Samples were labelled with AMAC, resolved by C18 RP-HPLC and detected by fluorescence. The numbers correspond to the elution positions of standard HS disaccharides. Peaks (a) and (b) corresponds to new HS products. Peak (*) corresponds to a minor AMAC-derived contaminant (note that most of excess free AMAC is strongly retained by the RP column and elutes later than AMAC-labelled disaccharides). Representative results of independent experiments conducted with two different cell preparations are shown.
(TIFF)

S2 Fig Comparison of the effects of HS3ST3A and HS3ST3B on colony formation and cell signaling in MDA-MB-231 cells. (**A**) Equal numbers of control and HS3ST-overexpressing cells (2000 per well) were seeded in six well plates and maintained for nine days in DMEM complemented with 1% FCS to form colonies. Fresh complete growth medium was then added for three days, after which the colonies were stained with crystal violet. The right panel represents the quantification of the colonies per well. Results are expressed as fold changes by comparison with control cells transfected with empty vector. Data are means ± S.D. from five separate experiments performed independently (***P < 0.001, significantly different when compared to control cells; *n*.*s*., not significantly different). **(B)** Twenty hours post transfection, MDA-MB-231 cells were serum-starved for 3 hours, collected and lysed. Proteins were then separated by SDS-PAGE and subjected to Western blotting with antibodies to I-κB, survivin, XIAP and to the phosphorylated forms of c-Src, Akt and NF-κB p65 subunit. Parallel immunoblotting with antibodies to GAPDH and to c-Src, Akt and NF-κB p65 regardless of their phosphorylation status confirmed equal loading of samples. Histograms represent the quantification of the phosphorylation status of c-Src, Akt, NF-κB p65 and of the expression of I-κB, survivin and XIAP related to GAPDH. Data were normalized to control cells. Representative results from three independent experiments are shown.
(TIFF)
