# Supplementary figures and images for: The heparan sulfate 3-O-sulfotransferases (HS3ST) 2, 3B and 4 enhance proliferation and survival in breast cancer MDA-MB-231 cells
Source: PLoS One. 2018 Mar 16;13(3):e0194676. doi: 10.1371/journal.pone.0194676 (PMC5856405; doi:10.1371/journal.pone.0194676)

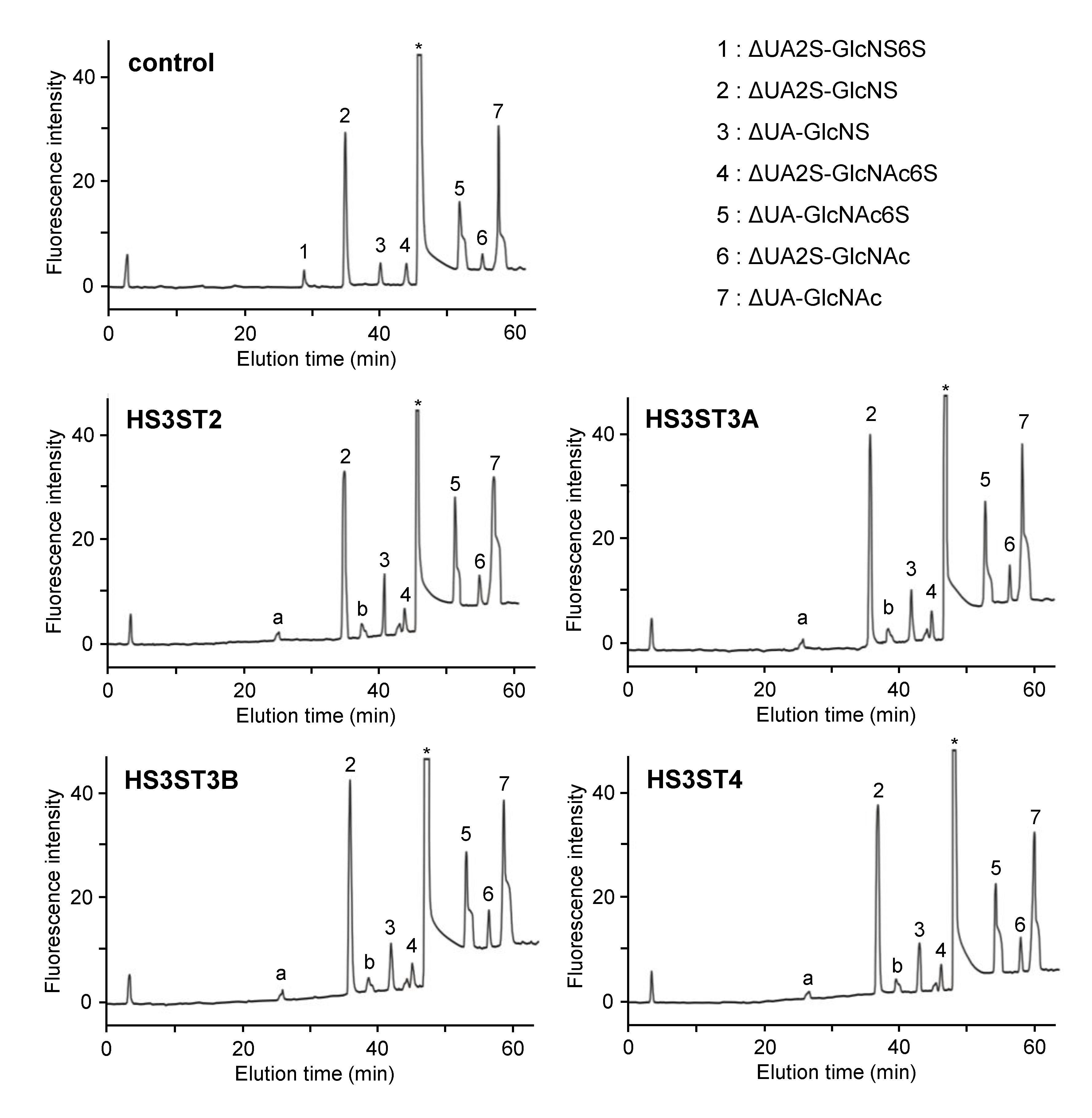

Supplement: S1 Fig — Purified HS from control (empty vector) and HS3ST-transfected cells were digested to disaccharides using a mixture of heparinases. Samples were labelled with AMAC, resolved by C18 RP-HPLC and detected by fluorescence. The numbers correspond to the elution positions of standard HS disaccharides. Peaks (a) and (b) corresponds to new HS products. Peak (*) corresponds to a minor AMAC-derived contaminant (note that most of excess free AMAC is strongly retained by the RP column and elutes later than AMAC-labelled disaccharides). Representative results of independent experiments conducted with two different cell preparations are shown. (TIFF) [file pone.0194676.s001.tiff]

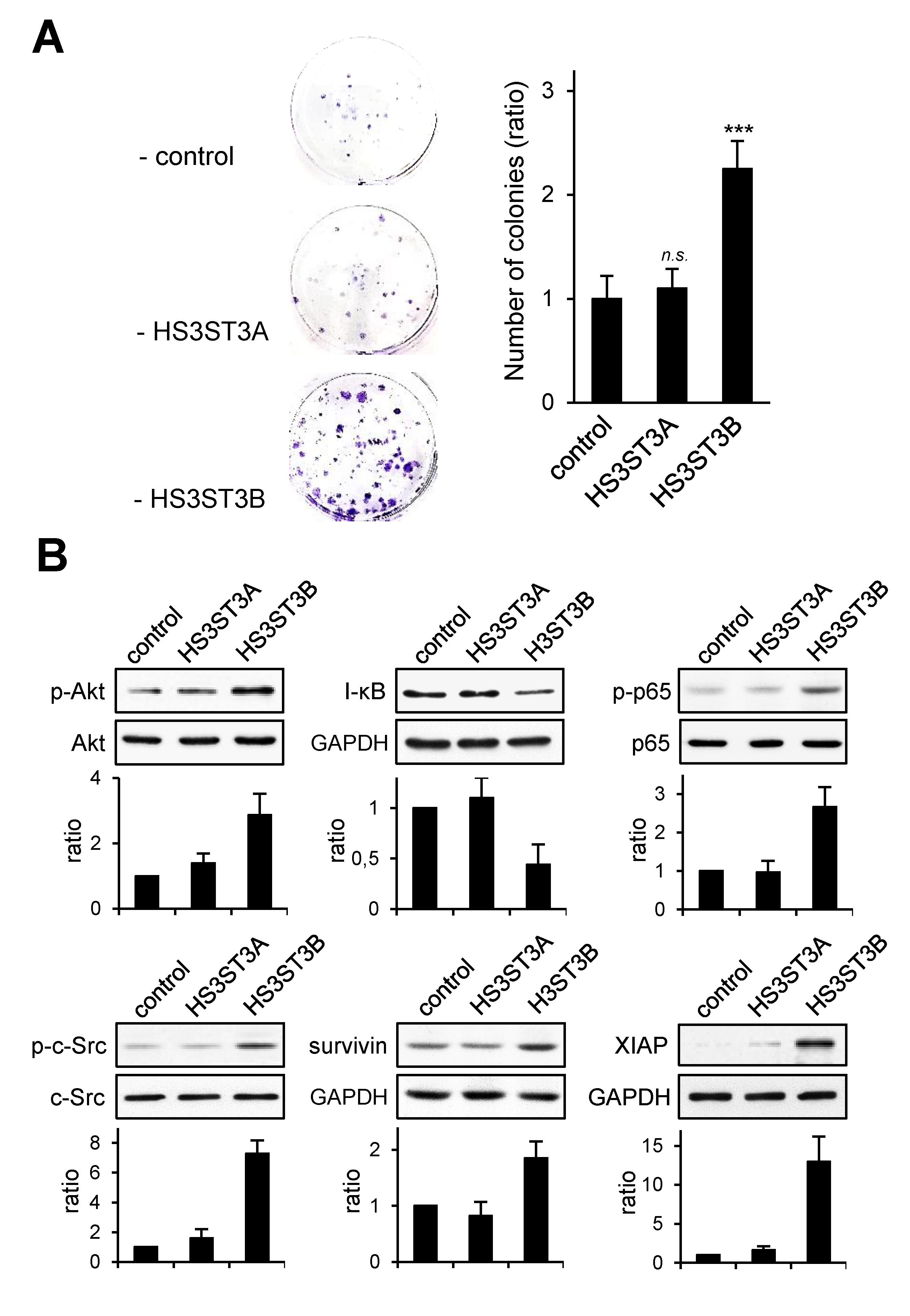

Supplement: S2 Fig — (A) Equal numbers of control and HS3ST-overexpressing cells (2000 per well) were seeded in six well plates and maintained for nine days in DMEM complemented with 1% FCS to form colonies. Fresh complete growth medium was then added for three days, after which the colonies were stained with crystal violet. The right panel represents the quantification of the colonies per well. Results are expressed as fold changes by comparison with control cells transfected with empty vector. Data are means ± S.D. from five separate experiments performed independently (***P < 0.001, significantly different when compared to control cells; n.s., not significantly different). (B) Twenty hours post transfection, MDA-MB-231 cells were serum-starved for 3 hours, collected and lysed. Proteins were then separated by SDS-PAGE and subjected to Western blotting with antibodies to I-κB, survivin, XIAP and to the phosphorylated forms of c-Src, Akt and NF-κB p65 subunit. Parallel immunoblotting with antibodies to GAPDH and to c-Src, Akt and NF-κB p65 regardless of their phosphorylation status confirmed equal loading of samples. Histograms represent the quantification of the phosphorylation status of c-Src, Akt, NF-κB p65 and of the expression of I-κB, survivin and XIAP related to GAPDH. Data were normalized to control cells. Representative results from three independent experiments are shown. (TIFF) [file pone.0194676.s002.tiff]
